# Low Interleukin-12 Levels concerning Severe Malaria: A Systematic Review and Meta-Analysis

**DOI:** 10.3390/ijerph19159345

**Published:** 2022-07-30

**Authors:** Polrat Wilairatana, Pattamaporn Kwankaew, Kwuntida Uthaisar Kotepui, Manas Kotepui

**Affiliations:** 1 Department of Clinical Tropical Medicine, Faculty of Tropical Medicine, Mahidol University, Bangkok 10400, Thailand; polrat.wil@mahidol.ac.th; 2 Medical Technology, School of Allied Health Sciences, Walailak University, Tha Sala, Nakhon Si Thammarat 80160, Thailand; pattamaporn.kw@wu.ac.th (P.K.); kwuntida.ut@wu.ac.th (K.U.K.)

**Keywords:** severe malaria, complicated malaria, interleukin-12, marker

## Abstract

Although many studies have investigated the role of interleukin (IL)-12 cytokine in the pathogenesis of severe malaria, these studies were based on a limited number of participants, possibly affecting their outcomes. We analyzed the difference in IL-12 levels between patients with severe and uncomplicated malaria through a meta-analysis. A systematic review was conducted following the Cochrane Handbook for Systematic Reviews of Interventions and was reported according to the Preferred Reporting Items for Systematic reviews and Meta-Analyses statement. Systematic literature searches were performed between 20 February and 2 March, 2022 in PubMed, Scopus, and Embase to identify studies reporting IL-12 levels in patients with severe and uncomplicated malaria. The quality of included studies was determined using the Strengthening the Reporting of Observational Studies in Epidemiology guidelines. The pooled mean difference (MD) in IL-12 between patients with severe and uncomplicated malaria was estimated using the DerSimonian–Laird method for the random-effects model. Altogether, 1885 potentially relevant articles were identified, and 10 studies enrolling 654 patients with severe malaria and 626 patients with uncomplicated malaria were included in the meta-analysis. Patients with severe malaria had lower mean IL-12 levels than those with uncomplicated malaria (*p* = 0.01, MD: −33.62, 95% confidence interval [CI]: −58.79 to −8.45, I^2^: 99.29%, 10 studies). In conclusion, decreased IL-12 levels might significantly contribute to the development of severe malaria. As most published literature demonstrated the role of IL-12 in animal models, human studies are required to understand the mechanisms involved in low IL-12 levels in patients with severe malaria.

## 1. Background

Malaria is caused by the infection of one of the six *Plasmodium* species, including *Plasmodium falciparum*, *Plasmodium vivax*, *Plasmodium malariae*, *Plasmodium ovale curtisi*, *Plasmodium ovale wallikeri*, and *Plasmodium knowlesi* [1]. There were an estimated 241 million malaria cases and 627,000 deaths in 2020 [2]. Severe falciparum malaria is defined as the presence of *P. falciparum* asexual parasitemia with one or more of the following complications including impaired consciousness, prostration, multiple convulsions, acidosis, hypoglycemia, severe malarial anemia, renal impairment, jaundice, pulmonary edema, significant bleeding, shock, and hyperparasitemia, as defined by the World Health Organization [3]. Malaria is mainly caused by *P. falciparum*; meanwhile, a lesser number of severe cases are caused by *P. vivax* [4], *P. knowlesi* [5], and other species [6,7]. Any patient with malaria who presented with severe malaria must be treated without delay according to the presence of complications, which would reduce the overall mortality rate to 10–20% [3].

Proinflammatory and anti-inflammatory cytokines mediate cellular immune responses to *Plasmodium* infection and contribute to malaria’s signs, symptoms, and pathophysiology [8]. Interleukin-12 (IL-12) is a 70 k-Da immunoregulatory cytokine produced mainly by antigen-presenting cells, including dendritic cells (DCs), neutrophils, macrophages, and human B-lymphoblastoid cells [9,10,11]. It plays a critical essential role in linking innate, non-specific, and adaptive immune, antigen-specific immunity [12]. During an intracellular infection, such as the presence of intracellular bacteria, intracellular parasite, and viral infection, the DC or other antigen-presenting cell migrates from the site of infection to a nearby lymph node where it presents the antigen to the naïve CD4+ T cell and releases IL-12 [12]. Once the antigen is recognized, the IL-12 induces the naïve T cell to become T helper type I (Th1). The primary function of the Th1 cell is to help macrophages fight against intracellular infections. After IL-12 stimulates the cells, Th1 starts secreting interferon-gamma (IFN-γ) and IL-2 [13]. The IFN-γ activates the macrophages to kill ingested bacteria and also stimulates antibody production, promoting the opsonization process to facilitate phagocytosis [14]. Concurrently, the secreted IL-2 enhances the growth and differentiation of other T cells [15]. Besides producing differentiated Th1 cells in response to antigen, IL-12 also induces the IFN-γ production from NK cells, which are also responsible for phagocyte activation, inflammation, and CD8+ T cell stimulation to eliminate the intracellular pathogen [10,11,16,17].

IL-12 might play a critical role in the adaptive immune response to malaria by promoting the proliferation of Th1 cells [18]. The cellular origins of IL-12 are DCs, neutrophils, and macrophages [19,20]. Monocytes have been linked to the pathogenesis of severe malaria via the production of damaging inflammatory cytokines that result in systemic inflammation and vascular dysfunction [21]. DCs serve as an essential link between innate and adaptive immunities [22]. DCs express co-stimulatory molecules necessary for the proliferation and differentiation of naïve T cells [22]. Previous research demonstrated that children infected with *P. falciparum* had fewer activated DCs during an acute, uncomplicated infection and had more DCs during a severe infection [23,24]. Neutrophils have been linked to adult and child mortality due to severe malaria [25,26]. Elevated IL-12 levels were suggested to play an essential role against systemic damage induced by malaria parasites [27]. Decreased IL-12 levels were related to thrombocytopenia in *P. vivax* malaria [28]. A previous study showed no difference in IL-12 levels between patients with malaria and non-malaria [29]. However, a previous study demonstrated that the IL-12 levels were elevated in all groups with malaria, indicating that IL-12 might be a predictor of acute malaria infection [30]. Although many studies have investigated the role of IL-12 cytokine in the pathogenesis of severe malaria, these studies were based on a limited number of participants, which could have affected the direction of the report’s outcome. Additionally, the role of IL-12 in severe and uncomplicated malaria has not yet been defined. To better understand the role of IL-12 in various clinical outcomes of patients with malaria, we analyzed the difference in IL-12 levels between patients with severe and uncomplicated malaria. Additionally, the difference in IL-12 levels between patients with uncomplicated malaria and healthy control participants was also analyzed.

## 2. Methods

### 2.1. Protocols

The systematic review and meta-analysis were conducted following the Cochrane Handbook for Systematic Reviews of Interventions [31]. Additionally, our systematic review and meta-analysis report followed the Preferred Reporting Items for Systematic reviews and Meta-Analyses (PRISMA) statement [32]. The systematic review was registered at PROSPERO (CRD42022315210).

### 2.2. Eligibility Criteria

We applied the PICO (P: participants, I: intervention, C: comparators, O: outcome) questions to design the eligibility criteria for study inclusion, which were as follows: (i) P: patients with severe malaria with any *Plasmodium* species; (ii) I: none; (iii) C: patients with non-severe or uncomplicated malaria; and (iv) O: IL-12 levels in patients with severe and uncomplicated malaria. The detection of malaria parasites could be performed using a rapid diagnosis test (RDT), microscopy, molecular techniques, or in combination. The quantification of IL-12 levels could be achieved using enzyme-linked immunosorbent assay (ELISA) or bead-based assay. We excluded the following studies: studies without full-text; studies with data on IL-12 levels in patients with severe malaria that cannot be extracted; studies with IL-12 levels in patients with severe malaria but only qualitative data were provided; in vitro studies; conference abstracts; studies with IL-12 levels measured in pregnancy/cord blood; studies reporting IL-12 levels in patients with severe malaria (n < 10, because these studies might report greater effect as compared to larger studies that may cause reporting biases); studies enrolling the same groups of participants; studies presenting IL-12 levels after treatment in malaria patients; non-English articles; and review articles.

### 2.3. Information Sources and Search Strategy

We conducted a systematic literature search in PubMed, Scopus, and Embase using the search combination “(malaria OR plasmodium OR plasmodia) AND (“Interleukin 12” OR IL12 OR IL-12 OR “Interleukin-12”) without limitation of publication date to obtain studies that documented IL-12 levels in patients with severe and uncomplicated malaria. The searches began on 20 February and ended on 2 March 2022, with the searches being limited to articles written in the English language (Table S1). The searches for relevant articles were also performed by reviewing the reference lists of the included studies and Google Scholar to ensure that the potentially relevant articles were not missed during the search processes.

### 2.4. Study Selection and Data Extraction

Two review authors (MK and KUK) were responsible for study selection independently. Disagreement between two authors during the study selection was resolved by discussion with another author (PW). Data of eligible studies were extracted independently by two authors (MK and PK) using a pre-prepared excel sheet. The following data were extracted from each study: author and year of publication, continents and country, study design, year of experiments, number and characteristics of participants, *Plasmodium* spp., age, male percentage, IL-12 levels (mean ± standard deviation [SD] or median with range in pg/mL), parasite density in parasites per microliter, method for detection of malaria parasites, and method for IL-12 quantification. Any inconsistency between the two authors was resolved by discussion for consensus.

### 2.5. Quality of the Included Studies

Two authors (MK and KUK) independently assessed the quality of included studies using the Strengthening the Reporting of Observational Studies in Epidemiology (STROBE) guidelines for cross-sectional studies, cohort/prospective observational studies, and case–control studies [33]. The tools contain 22 items based on the following data: title and abstract, introduction, methods, participants, statistical methods, results, discussion, and other information. Every single item was scored one score per item with 22 scores. The quality of each study was rated as low, moderate, and high quality by percentile scores of <0, 50–75, and ≥75, respectively.

### 2.6. Effect Measures

The primary effect measure was the pooled mean difference (MD) of IL-12 between patients with severe and uncomplicated malaria. The secondary effect measure was the pooled MD of IL-12 between patients with uncomplicated malaria and healthy control participants.

### 2.7. Synthesis Methods

The mean and standard deviation (SD) of IL-12 levels from two groups of patients from each study were filled in an excel sheet before being imported too Stata software version 17.0 (StataCorp LLC, College Station, TX, USA). If the SD of IL-12 levels was missing from the included studies, the SD of IL-12 levels was borrowed from the study reporting a similar mean, as described previously [31]. The mean and SD were calculated if the study reported the median and range of IL-12 levels, as described previously [34]. The meta-analyses were carried out using meta written command in Stata software. We used the DerSimonian–Laird method for the random-effects model to synthesize the pooled MD, 95% confidence interval (CI), and weighted from each study. Cochrane Q and inconsistency index (I^2^ statistics) were used to assess the heterogeneity of the effect estimate among the included studies. Cochrane Q with a *p* < 0.1 indicated a significant heterogeneity of effect estimates among the included studies. I^2^ values < 25%, 25–75%, and >75% were considered as low, moderate, and high levels of heterogeneity, respectively. We have conducted meta-regression and subgroup analyses using the characteristics of the included studies to identify the source(s) of heterogeneity of the effect estimates among the included studies.

### 2.8. Reporting Bias Assessment

The publication bias was assessed by visual inspection of the funnel plot asymmetry and validated using Egger’s test. The cause of funnel plot asymmetry was further explored using the contour-enhanced funnel plot. If publication bias was presented, the trim and fill method was applied to adjust the pooled effect estimates.

### 2.9. Certainty Assessment

The leave-one-out sensitivity analysis was performed to assess whether a single study did not affect the overall pooled effect estimate.

## 3. Results

### 3.1. Search Results

**Figure 1:**
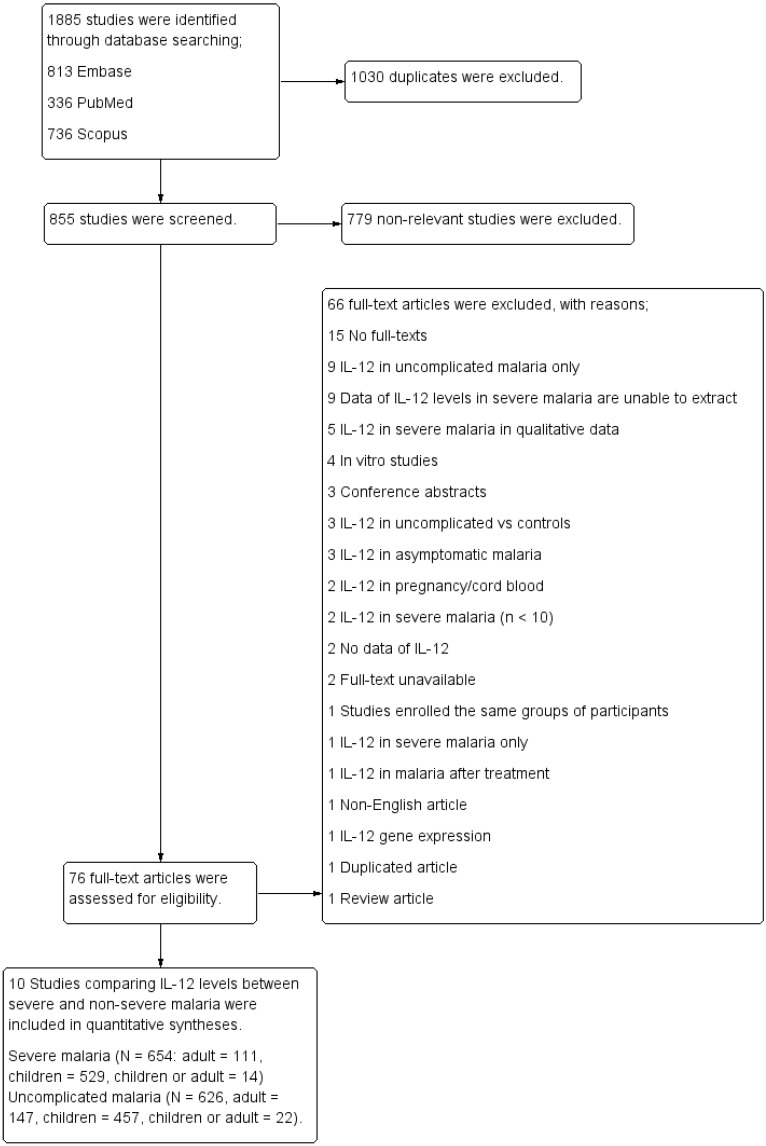
Study flow diagram.

A total of 1885 articles were identified through database searching (813 from Embase, 336 from PubMed, and 736 articles from Scopus). After 1030 duplicates were excluded, the titles and abstracts of the remaining 855 articles were screened. After 779 non-relevant articles were excluded, the remaining 76 full-text articles were assessed for eligibility, and 66 full-text articles were excluded owing to the following reasons: 15 with no full-texts; nine presenting IL-12 levels in cases of uncomplicated malaria only; nine studies wherein the data on IL-12 levels in patients with severe malaria cannot be extracted; five studies describing the IL-12 levels in patients with severe malaria in qualitative data (no mean or median); four were in vitro studies; three were conference abstracts with incomplete data; three reported IL-12 levels of patients with uncomplicated malaria and controls; three reported IL-12 levels of patients with asymptomatic malaria; two presenting the levels of IL-12 in pregnancy or cord blood; two presenting IL-12 levels in patients with severe malaria (n < 10); two without data on IL-12; two with unavailable full-texts; one enrolling the same groups of participants; one presenting IL-12 of patients with severe malaria only; one presenting IL-12 after treatment in patients with malaria; one was s non-English article; one reported on IL-12 gene expression; one was a duplicated article; and one was a review article. Finally, ten studies [35,36,37,38,39,40,41,42,43,44] that compared the IL-12 levels between patients with severe and non-severe malaria were included in the quantitative syntheses (Figure 1).

### 3.2. Characteristics of the Included Studies

The included studies were published between the years 2000 and 2010 (Table 1). Most of the included studies (5/10, 50%) were case–control [39,40,41,42,43] and prospective observational (4/10, 40%) studies [35,36,37,38]. Most of the included studies were conducted in Africa (6/10, 60%) [35,36,39,41,42,44] and Asia (2/10, 20%) [37,43]. Most studies enrolled patients with *P. falciparum* infection (8/10, 80%) [35,36,39,40,41,42,43,44] and children (6/10, 60%) [35,36,39,41,42,44]. The IL-12 levels between patients with severe and uncomplicated malaria were available for analysis in 10 studies; meanwhile, the data on the IL-12 levels between patients with uncomplicated malaria and healthy controls were available for analysis in six studies [36,39,40,41,42,43]. Most studies performed the microscopic method for the identification of malaria parasites (8/10, 80%) [35,36,37,39,40,41,42,44]; meanwhile, two studies [38,43] performed more than two methods for the identification of malaria parasites. Most of the included studies (9/10, 90%) used ELISA for quantification of IL-12 levels [35,36,37,38,39,40,42,43,44]; meanwhile, only one study [41] used bead-based assay for quantification. Seven studies (7/10, 70%) investigated the IL-12 levels without defining the IL-12 subunit [36,37,38,39,42,43,44], whereas, two studies (2/10, 20%) investigated the IL-12p70 levels [40,41] and one study specified the IL-12 subunit (p70 heterodimer and p40 chain) [35].

### 3.3. Quality of the Included Studies

The quality of the included studies was assessed using the STROBE Checklist. Nine studies were of high quality [35,36,38,39,40,41,42,43,44] whereas, only one study was of moderate quality [37]. No study was excluded; therefore, 10 studies were included in the meta-analysis.

### 3.4. IL-12 in Severe and Uncomplicated Malaria

**Figure 2:**
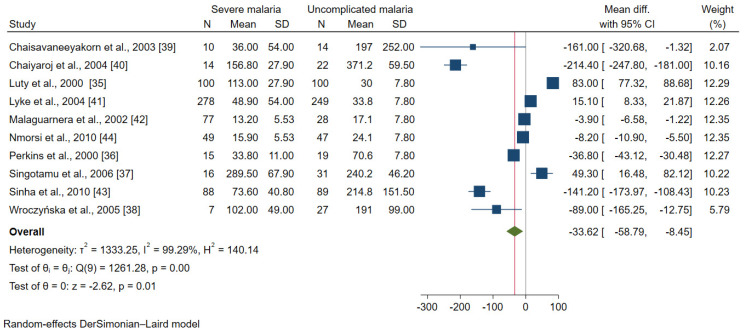
Forrest plot demonstrated the difference in the mean IL-12 levels (pg/mL) between patients with severe malaria and uncomplicated malaria [35,36,37,38,39,40,41,42,43,44]. Abbreviation: Mean Diff., mean difference; CI, confidence interval. Explanation of the forest plot: squared-box symbol, point estimate; green diamond and red line: pooled mean difference: blue line: line of no effect; I^2^, level of heterogeneity; *p* = 0.00 or less than 0.05, significant heterogeneity.

Data on the IL-12 levels between patients with severe malaria (654 cases) and uncomplicated malaria (626 cases) were available for analysis in 10 studies [35,36,37,38,39,40,41,42,43,44]. The results of the individual study showed that seven studies demonstrated lower mean IL-12 levels in patients with severe malaria than in those with uncomplicated malaria [36,38,39,40,42,43,44]. Meanwhile, three studies demonstrated higher mean IL-12 levels in patients with severe malaria than in those with uncomplicated malaria [35,37,41]. Overall, the meta-analysis results showed lower mean IL-12 levels in patients with severe malaria than in those with uncomplicated malaria (*p* = 0.01, MD: −33.62, 95% CI: −58.79 to −8.45, I^2^: 99.29%, 10 studies, Figure 2).

**Figure 3:**
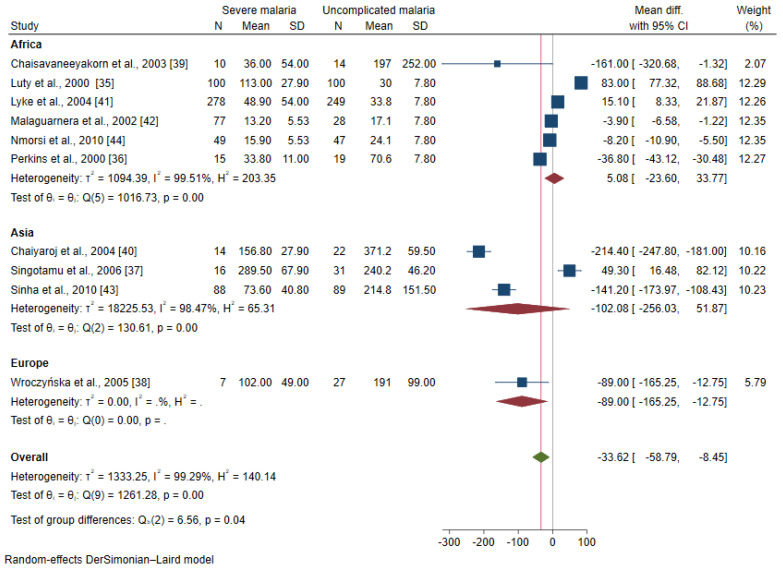
Forrest plot demonstrated the difference in the mean IL-12 levels (pg/mL) between patients with severe malaria and uncomplicated malaria stratified by continents [35,36,37,38,39,40,41,42,43,44]. Abbreviation: Mean Diff., mean difference; CI, confidence interval. Explanation of the forest plot: squared-box symbol, point estimate; green diamond and red line: pooled mean difference: red diamond: pooled mean difference in each subgroup; blue line: line of no effect; I^2^, level of heterogeneity; *p* = 0.00 or less than 0.05, significant heterogeneity.

**Figure 4:**
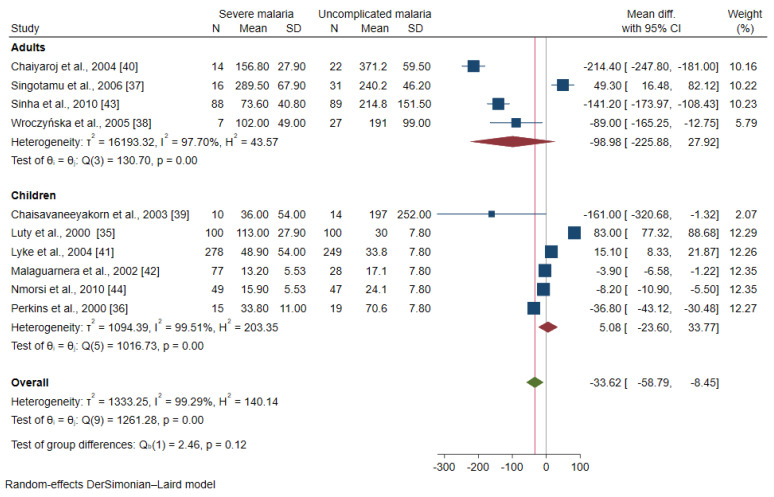
Forrest plot demonstrated the difference in the mean IL-12 levels (pg/mL) between patients with severe malaria and uncomplicated malaria stratified by age groups [35,36,37,38,39,40,41,42,43]. Abbreviation: Mean Diff., mean difference; CI, confidence interval. Explanation of the forest plot: squared-box symbol, point estimate; green diamond and red line: pooled mean difference: red diamond: pooled mean difference in each subgroup; blue line: line of no effect; I^2^, level of heterogeneity; *p* = 0.00 or less than 0.05, significant heterogeneity.

The meta-regression analysis using continents and age groups as covariates demonstrated that these covariates were confounding the effect estimate (pooled MD) of the included studies (*p* < 0.0001). A subgroup analysis of continents and age groups was further performed. The subgroup analysis of the continents showed a subgroup difference (*p* = 0.04). No difference in the mean IL-12 level was observed between patients with severe malaria and uncomplicated malaria among studies conducted in Africa (MD: 5.08, 95% CI: −23.6–33.77, I^2^: 99.51%, six studies) and Asia (MD: −102.08, 95% CI: −256.03–51.87, I^2^: 98.5%, three studies, Figure 3). The subgroup analysis of age groups showed no subgroup difference (*p* = 0.12). No difference in the mean IL-12 levels between patients with severe malaria and uncomplicated malaria was observed among studies enrolling children (MD: 5.08, 95% CI: −23.6–33.77, I^2^: 99.51%, six studies) and adults (MD: −98.98, 95% CI: −225.88–27.92, I^2^: 97.70%, four studies, Figure 4).

For the IL-12 levels in patients infected by different *Plasmodium* spp., two studies enrolled patients with *P. falciparum* [35,41] and demonstrated higher mean IL-12 levels in patients with severe malaria than in those with uncomplicated malaria. Meanwhile, six studies enrolling patients with *P. falciparum* demonstrated lower mean IL-12 levels in those with severe malaria than in those with uncomplicated malaria [36,39,40,42,43,44]. Singotamu et al. [37] showed higher mean IL-12 levels in patients with severe *P. vivax* malaria than in those with uncomplicated *P. vivax* malaria (MD: 49.30, 95% CI: 16.48–82.12). Wroczyńska et al. [38] who enrolled patients with *P. falciparum*, *P. vivax*, *P. ovale*, and *P. malariae* showed lower mean IL-12 levels in those with severe malaria (*P. falciparum*) than in those with uncomplicated malaria.

### 3.5. IL-12 in Patients with Uncomplicated Malaria and Healthy Controls

**Figure 5:**
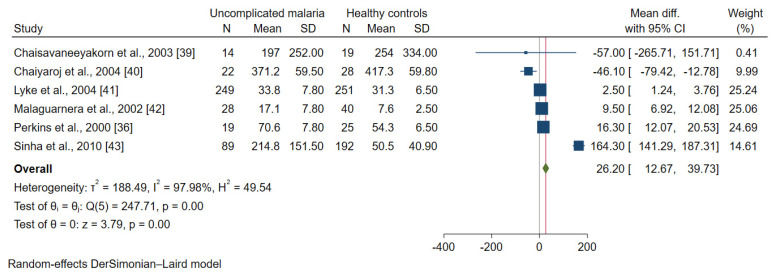
Forrest plot demonstrated the difference in the mean IL-12 levels (pg/mL) between patients with uncomplicated malaria and healthy controls [36,39,40,41,42,43]. Abbreviation: Mean Diff., mean difference; CI, confidence interval. Explanation of the forest plot: squared-box symbol, point estimate; green diamond and red line: pooled mean difference: blue line: line of no effect; I^2^, level of heterogeneity; *p* = 0.00 or less than 0.05, significant heterogeneity.

Data on the IL-12 levels between patients with uncomplicated malaria (421 cases) and healthy controls (555 cases) were available for analysis in six studies [36,39,40,41,42,43]. Only one study demonstrated a lower mean IL-12 level in patients with uncomplicated malaria than in healthy controls [40]. Meanwhile, four studies demonstrated higher mean IL-12 levels in patients with uncomplicated malaria than in healthy controls [36,41,42,43]. Overall, the meta-analysis results showed higher mean IL-12 levels in patients with uncomplicated malaria than in healthy controls (*p* = 0.01, MD: 26.20, 95% CI: 12.67–39.73, I^2^: 97.98%, six studies, Figure 5).

**Figure 6:**
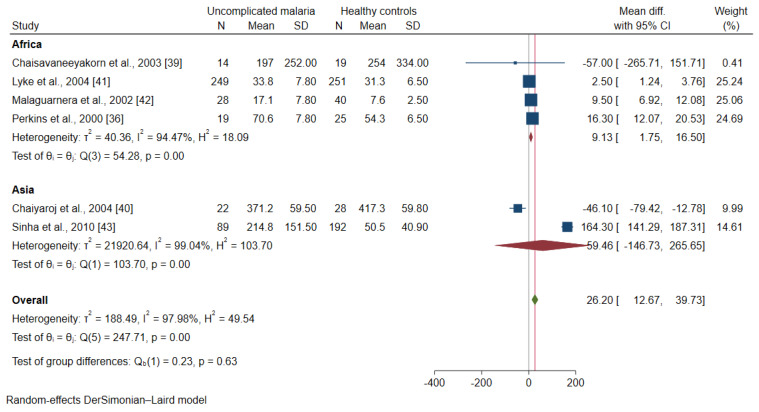
Forrest plot demonstrated the difference in the mean IL-12 levels (pg/mL) between patients with uncomplicated malaria and healthy controls by continents [36,39,40,41,42,43]. Abbreviation: Mean Diff., mean difference; CI, confidence interval. Explanation of the forest plot: squared-box symbol, point estimate; green diamond and red line: pooled mean difference: red diamond: pooled mean difference in each subgroup; blue line: line of no effect; I^2^, level of heterogeneity; *p* = 0.00 or less than 0.05, significant heterogeneity.

**Figure 7:**
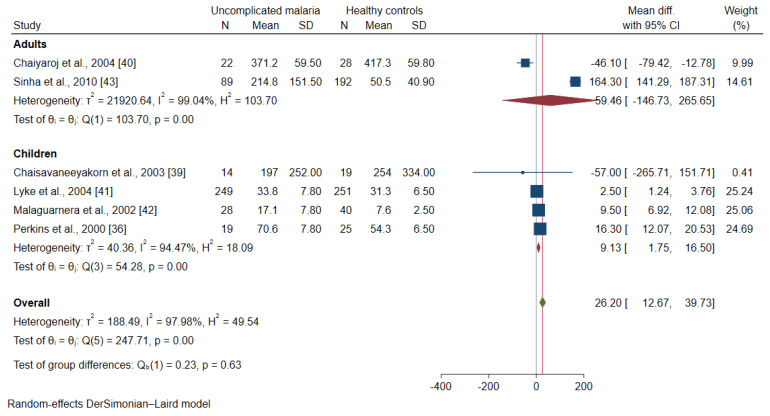
Forrest plot demonstrated the difference in the mean IL-12 levels (pg/mL) between patients with uncomplicated malaria and healthy controls by age groups [36,39,40,41,42,43]. Abbreviation: Mean Diff., mean difference; CI, confidence interval. Explanation of the forest plot: squared-box symbol, point estimate; green diamond and red line: pooled mean difference: red diamond: pooled mean difference in each subgroup; blue line: line of no effect; I^2^, level of heterogeneity; *p* = 0.00 or less than 0.05, significant heterogeneity.

The meta-regression analysis using continents and age groups as covariates demonstrated that these covariates were confounding the effect estimate (pooled MD) of the included studies (*p* < 0.001). A subgroup analysis of continents and age groups was further performed. The subgroup analysis of continents showed a subgroup difference (*p*-value = 0.63). Patients with uncomplicated malaria had higher mean IL-12 levels than healthy controls among studies conducted in Africa (MD: 9.13, 95% CI: 1.75–16.5, I^2^: 94.5%, four studies). Meanwhile, no difference in the mean IL-12 levels was found between patients with uncomplicated malaria and healthy controls among studies conducted in Asia (MD: 59.46, 95% CI: −146.7–265.7, I^2^: 99%, two studies, Figure 6). A subgroup analysis of age groups showed no subgroup difference (*p* = 0.63). Patients with uncomplicated malaria had higher mean IL-12 levels than healthy controls among studies enrolling children (MD: 9.13, 95% CI: 1.75–16.50, I^2^: 94.5%, four studies). Meanwhile, no difference in the mean IL-12 levels was observed between patients with uncomplicated malaria and healthy controls among studies enrolling adults (MD: 59.46, 95% CI: −146.7–265.7, I^2^: 94.5%, two studies, Figure 7).

### 3.6. Sensitivity Analysis

**Figure 8:**
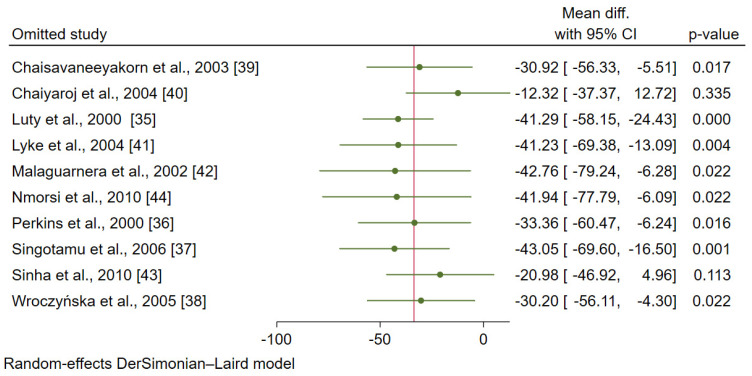
The results of the leave-one-out meta-analysis of the difference in the mean IL-12 levels (pg/mL) between patients with severe malaria and uncomplicated malaria [35,36,37,38,39,40,41,42,43,44].

A sensitivity analysis was performed using a leave-one-out technique to determine whether leaving each study might affect the pooled estimate (MD). After leaving eight studies [35,36,37,38,39,41,42,44], the meta-analysis results demonstrated a difference in the mean oIL-12 levels between patients with severe and uncomplicated malaria (*p* < 0.05, Figure 8). Meanwhile, after leaving two studies [40,43], the meta-analysis results demonstrated no difference in the mean IL-12 levels between patients with severe and uncomplicated malaria (*p* > 0.05).

**Figure 9:**
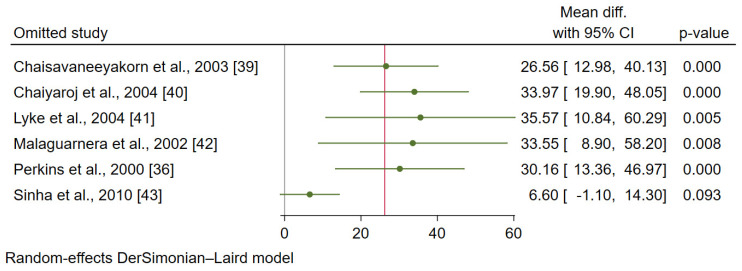
The results of the leave-one-out meta-analysis of the difference in the mean IL-12 levels (pg/mL) between patients with uncomplicated malaria and healthy controls [36,39,40,41,42,43].

After leaving five studies (one-by one) and re-run the meta-analysis [36,39,40,41,42], the meta-analysis results demonstrated the difference in the mean IL-12 levels between patients with uncomplicated malaria and healthy controls (*p* < 0.05, Figure 9). Meanwhile, after leaving one study and re-run the meta-analysis [43], the meta-analysis results demonstrated no difference in the mean IL-12 levels between patients with uncomplicated malaria and healthy controls (*p* > 0.05).

### 3.7. Publication Bias

**Figure 10:**
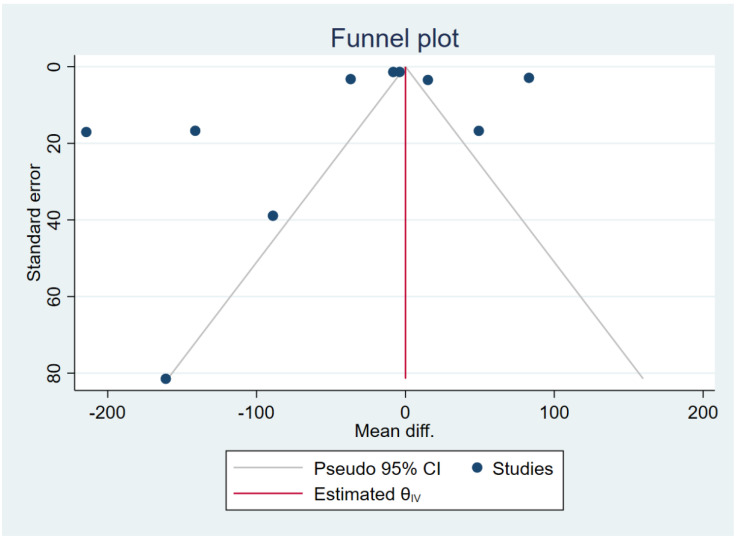
The funnel plot demonstrated the distribution of the mean difference of IL-12 levels between severe and uncomplicated malaria from each study. In addition, the funnel plots showed the asymmetrical distribution of the mean differences and the standard error (se) of the mean differences.

**Figure 11:**
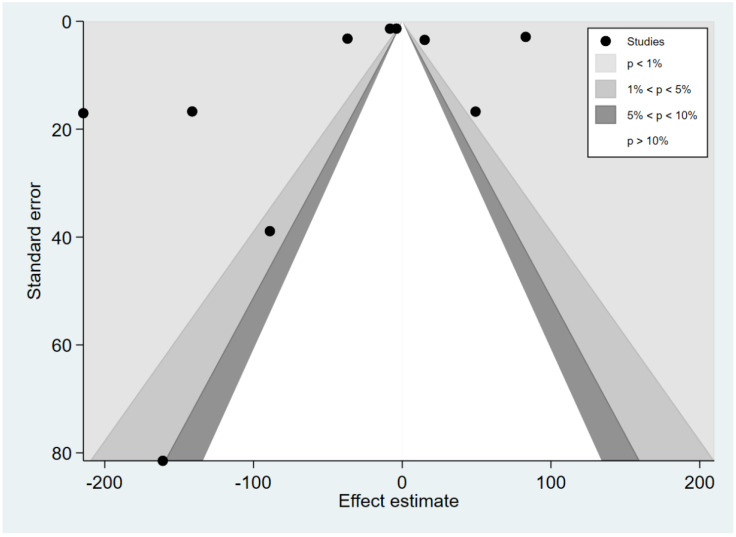
Contour-enhanced funnel plot demonstrated the distribution of the mean difference of IL-12 levels between severe and uncomplicated malaria from each study. In addition, the funnel plots showed the distribution of the mean differences and the standard error (se) of the mean differences in both significant and non-significant areas.

**Figure 12:**
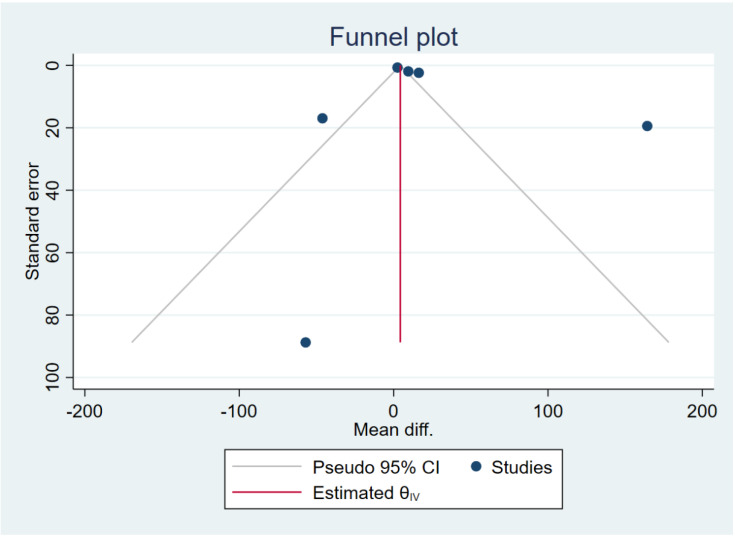
The funnel plot demonstrated the mean difference of IL-12 levels between uncomplicated malaria and healthy controls from each study. In addition, the funnel plots showed the asymmetrical distribution of the mean differences and the standard error (se) of the mean differences.

**Figure 13:**
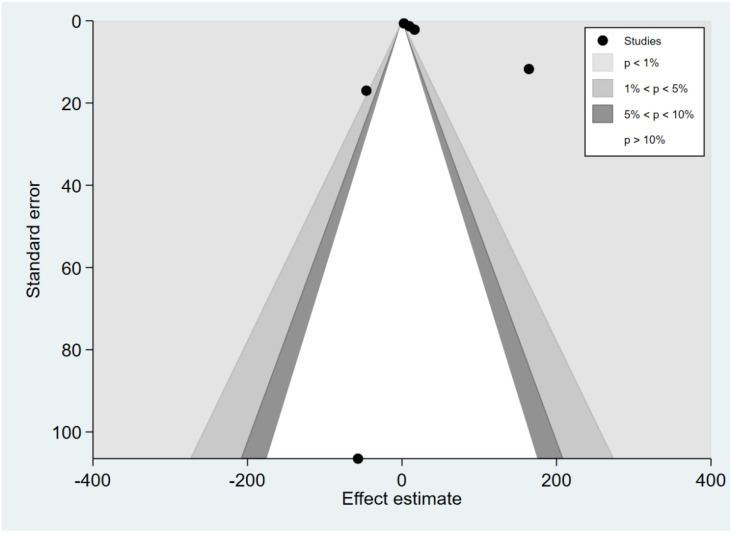
Contour-enhanced funnel plot demonstrated the distribution of the mean difference of IL-12 levels between uncomplicated malaria and healthy controls from each study. In addition, the funnel plots showed the distribution of the mean differences and the standard error (se) of the mean differences in both significant and non-significant areas.

The funnel plot was asymmetrical for the difference in the mean IL-12 levels between patients with severe and uncomplicated malaria (Figure 10). Egger’s test demonstrated a small-study effect (*p* = 0.0008). The contour-enhanced funnel plot demonstrated that effect estimates (MD) were located in the significant areas (*p* < 0.05), indicating that the cause of funnel plot asymmetry was publication bias (Figure 11). The trim and fill method had been applied to correct the pooled effect estimate. The results showed no difference in the IL-12 levels between patients with severe and uncomplicated malaria after adjusting for publication (pooled MD: 0.04, 95% CI: −1.64–1.71). The funnel plot was asymmetrical for the difference in the mean IL-12 levels between patients with uncomplicated malaria and healthy controls (Figure 12). Egger’s test demonstrated no small-study effect (*p* = 0.19). Contour-enhanced funnel plot demonstrated that most of the effect estimates (MD) were located in the significant areas (*p* < 0.05), indicating that the cause of funnel plot asymmetry was publication bias (Figure 13). The trim and fill method had been applied to correct the pooled effect estimate. The results showed that the IL-12 levels in patients with uncomplicated malaria than healthy controls after adjusting for publication bias (pooled MD: 4.98, 95% CI: 3.89–6.07).

## 4. Discussion

Several cytokines have been reported in association with malaria severity. In the present study, data on the IL-12 levels in patients with severe and uncomplicated malaria were collated and quantitatively synthesized through a meta-analysis approach. The meta-analysis results showed that patients with severe malaria had lower IL-12 levels than those with uncomplicated malaria, indicating that IL-12 might be a candidate marker for severe malaria.

Impaired IL-12 production in patients with severe malaria but not in uncomplicated malaria [35], particularly with hyperparasitemia, was associated with reduced IFN-γ levels [30]. The previous study also showed that TGF-β downregulates the IL-12 levels, thereby modulating the immune response to *P. falciparum*, preventing patients from developing severe diseases, such as cerebral malaria and severe anemia [45]. In the gene expression analysis, elevated levels of TNF-α and IFN-γ with downregulation of IL-2 and upregulation of TGF-β mRNA levels were observed in patients with severe malaria, but not in patients with uncomplicated malaria [46]. The study in patients with brain swelling demonstrated a higher IL-12 level in patients with severe and moderate brain swelling than in those without brain swelling, indicating that IL-12 was a marker for severe disease [47]. The IL-12 levels were reported to be lower in patients with severe malaria [36,42], particularly in those with severe malarial anemia, because the ingestion of malarial pigments by monocytes promoted the overproduction of IL-10, TNF-α, or TGF-β, and caused a lower production of IL-12 [36,48].

Increased IL-12 levels in patients with severe malaria were also reported previously, and its elevation was not correlated with parasitemia [49]. Three studies included in the meta-analysis demonstrated a higher mean IL-12 level in patients with severe malaria than in those with uncomplicated malaria [35,37,41]. The study by Luty et al. explained that increased IL-12 levels were associated with anemia severity [35]; thereby, IL-12 might promote hemoglobin production and maintain appropriate hemoglobin levels in patients with malaria [50]. Meanwhile, another study by Lyke et al. proposed that the slight increase in the IL-12 levels may be the result of downregulation by IL-10 [41]. The study by Singotamu et al. reported slightly increased IL-12 levels and significantly increased IL-10 and TNF-α levels in patients with severe malaria [37]. Low IL-12 levels were associated with hyperlactatemia in patients with severe malarial anemia [51]. In addition, increased IL-12 levels were associated with anemia severity [35]; thereby, IL-12 might promote hemoglobin production and maintain appropriate hemoglobin levels in patients with malaria [50]. Furthermore, IL-12 and IL-18 synergistically could increase the IFN-γ production by macrophages, T cells, and NK cells, indicating that IL-12 plays a role in cell-mediated immune response in malaria [52,53].

There was an argument about the potential of IL-12 as a candidate for predicting severe malaria. The previous study suggested that serum IL-12 levels and other cytokines such as TNF-α, IFN-γ, and CRP levels might not be candidate markers for severe malaria because of the absence in the difference in their levels in higher parasite densities [54]. Additionally, increased IL-12 levels were reported in patients with severe malaria but it was not correlated with parasitemia [49]. Nevertheless, the strong inverse correlation between IL-12 levels and *P. falciparum* parasitemia had been reported previously [35]. A protective role of IL-12 for severe malaria was previously reported; IL-12, in combination with inducible nitric oxide synthase (iNOS), could improve the oxygen delivery in the microcirculation of patients with severe malaria [51], thereby protecting the pathogenesis of severe *P. falciparum* malaria. From these points of view, the dysregulation of cytokine networks rather than a single cytokine involves severe malaria pathogenesis.

Age might be confounding the difference in the IL-12 levels between patients with severe and uncomplicated malaria, which might be attributed to the age-dependent acquisition of immunity. Increased IL-12 levels indicated that younger patients had a higher innate immune response than older adults due to an age-differential reaction to Toll-like receptors (TLRs) [55,56]. Therefore, adult patients might be at a lower risk of severe malaria than younger children [57]. However, the subgroup analysis of age indicated that age was not a confounder of IL-12 levels, suggesting the need for further studies to consider age in the cytokine studies. In the subgroup analysis of continents, higher mean IL-12 levels were observed in patients with uncomplicated malaria than in healthy controls from Africa; meanwhile, there was no difference in the IL-12 levels between patients with uncomplicated malaria and healthy controls among studies conducted in Asia. These results could be explained by the fact that studies conducted in Africa enrolled children whereas those conducted in Asia enrolled adults. This difference could also be explained by the results of the subgroup analysis of age showing higher mean IL-12 levels in children with uncomplicated malaria than in healthy controls. Meanwhile, no difference in the mean IL-12 levels was found between adults with uncomplicated malaria and healthy controls.

Increased IL-12 levels were found in patients with acute uncomplicated malaria but not in healthy controls. In the meta-analysis, four studies [36,41,42,43] reported increased IL-12 levels in patients with uncomplicated malaria, as compared to healthy controls. This result indicated that the IL-12 level was the marker of acute malaria infection. High IL-12 levels during the acute phase of an uncomplicated *P. falciparum* infection indicate the early and effective immune response by the proinflammatory Th1 cytokines [58]. Another study demonstrated that the IL-12 levels were increased on day 0 and continuously decreased from day 0 to day 10 [59]. The increased IL-12 levels during the first day of admission might indicate that IL-12 modulates macrophage activity as the first line of defense against malaria infection, increasing erythrocyte destruction and bone marrow dyserythropoiesis [60]. The increased IL-12 levels in patients with uncomplicated malaria were related to the increased TNF-α levels; TNF-α is an essential cofactor for IL-12 that induces the NK cells to produce interferon γ [30]. Therefore, the IL-12 levels were increased between days 2 and 3 of treatment due to decreased IL-10 levels [61]. Therefore, detection of IL-12 in the acute phase of infection in the first week could be used as a candidate marker for malaria infection. In the returning travelers, IL-12 levels were reported to be increased in patients with severe malaria, which might also be indicative of worse outcomes in these patients [62].

Rather than a candidate marker for malaria infection, IL-12 might be used to differentiate malaria from other tropical diseases. For example, a previous study found that the IL-12 levels were significantly higher in patients with *P. falciparum* than in those with Chikungunya virus (CHIKV) [63]. Another study demonstrated that the IL-12 levels were significantly higher in patients with *P. falciparum* than in those with dengue virus (DENV) and those with malaria-dengue co-infection, indicating that IL-12 was the marker of acute malaria infection [64]. Besides uncomplicated malaria, no difference in the IL-12 levels between patients with asymptomatic malaria and non-infected individuals was observed in a previous study [65]. An in vitro study demonstrated that the expression of IL-12 was related to the absence of parasitemia in asymptomatic malaria [66]. However, no significant correlation was found between the IL-12 levels and parasitemia levels among asymptomatic individuals [67]. The production of IL-12 enhanced the IL-6 production, followed by an increase in IL-10 production, to inhibit the production of TNF-α, which is a proinflammatory cytokine [29].

Most of the mechanistic role of IL-12 in malaria came from a mouse model of experimental malaria infection. A previous study showed that antibody-mediated protective immunity against blood-stage *P. chabaudi* required IL-12 [68]. IFN-γ and TNF-α were required for IL-12-induced protection against blood-stage *P. chabaudi* AS, which occurred via a nitric oxide-dependent mechanism [69]. Moreover, treatment with a low dose of IL-12 and chloroquine completely cured blood-stage malaria, prevented severe anemia, and induced immunity to reinfection in a mice model [70].

The present study had limitations. First, the number of included studies for meta-analysis was limited. Second, although the searches had been performed in Google Scholar as an additional source; unfortunately, no eligible article was found. Google Scholar does not support many of the features required for systematic searches [60]; it lacks truncation, proximity operators, use of parentheses, and search history [61]. There is also a limitation in the usability of Google Scholar for medical research purposes [62]. Third, although we identified two sources of heterogeneity of the outcome among the included studies, there might be other sources of heterogeneity that are yet to be identified. Fourth, a publication bias among the included studies might affect the pooled effect estimate. However, our analyses were performed with adjustment for publication bias, and the adjusted effect estimate was presented in this study. Fifth, most of the included studies enrolled patients with *P. falciparum* infections (80%), and only one study enrolled patients with *P. vivax* malaria [37]. Therefore, the meta-analysis could not determine the difference in the IL-12 level among the *Plasmodium* species. Sixth, the exact time of the increase in IL-12 levels and return to normal levels in severe malaria patients was not mentioned. Lastly, the present systematic review included only articles written in the English language; therefore, articles written in other languages, such as French and Chinese, might have been missed.

## 5. Conclusions

In summary, the present systematic review and meta-analysis demonstrated that decreased IL-12 levels might significantly contribute to the development of severe malaria. As most of the studies investigated the role of IL-12 in animal models, further studies in humans are required to understand the mechanisms involved in low IL-12 levels among patients with severe malaria.

## Figures and Tables

**Table 1:** Characteristics of the included studies.

Characteristics	n	%	References
**Study designs**			
Prospective observational studies	4	40	[35,36,37,38]
Case–control studies	5	50	[39,40,41,42,43]
Cross-sectional studies	1	10	[44]
**Study areas**			
Africa	6	60	[35,36,39,41,42,44]
Asia	2	20	[37,43]
Asia and Africa	1	10	[40]
Europe	1	10	[38]
***Plasmodium* spp** **.**			
*P* *. falciparum*	8	80	[35,36,39,40,41,42,43,44]
*P* *. vivax*	1	10	[37]
*P* *. falciparum* */P* *. vivax* */P* *. ovale* */P* *. malariae*	1	10	[38]
**Participants**			
Children	6	60	[35,36,39,41,42,44]
Adults	3	30	[37,38,43]
All age groups	1	10	[40]
**Methods** **of malaria detection**			
Microscopy	8	80	[35,36,37,39,40,41,42,44]
Microscopy/RDT/PCR	1	10	[43]
Microscopy/PCR/IFA	1	10	[38]
**Methods for IL** **-12 quantification**			
ELISA	9	90	[35,36,37,38,39,40,42,43,44]
BD Pharmingen			[39]
R&D Systems			[36,40,42]
BD Biosciences			[41,43]
BioSource			[35]
Abcam			[44]
Diaclone			[37]
BenderMedSystems			[38]
Bead-based assay (BD Biosciences)	1	10	[41]
**IL-12 subunit**			
IL-12 (subunit not defined)	7	70	[36,37,38,39,42,43,44]
IL-12 (p70 heterodimer and p40 chain)	1	10	[35]
IL-12p70	2	20	[40,41]

Abbreviation: ELISA, Enzyme-linked immunosorbent assay; IFA, Indirect fluorescent antibody test; PCR, Polymerase chain reaction; RDT, Rapid diagnostic tests.

## Data Availability

All data and related materials are presented in this manuscript.

## Supplementary Materials

The following supporting information can be downloaded at: https://www.mdpi.com/article/10.3390/ijerph19159345/s1, Table S1: Search terms; Table S2: Details of the included studies; Table S3: Quality of the included studies. PRISMA_2020_checklist. PRISMA_2020_abstract_checklist.

## Abbreviations

CI: Confidence interval; DENV: Dengue virus; ELISA: Enzyme-linked immunosorbent assay; IL-12: Interleukin-12; MD: Mean difference; iNOS: Nitric oxide synthase; PRISMA: Preferred Reporting Items for Systematic reviews and Meta-Analyses; RDT: Rapid diagnosis test; SD: Standard deviation; STROBE: Strengthening the Reporting of Observational Studies in Epidemiology; Th: T helper type I; WHO: World Health Organization.
