# Supplementary material for: Low Interleukin-12 Levels concerning Severe Malaria: A Systematic Review and Meta-Analysis
Source: Int J Environ Res Public Health. 2022 Jul 30;19(15):9345. doi: 10.3390/ijerph19159345 (PMC9368085; doi:10.3390/ijerph19159345)
Supplement: Supplementary file 1 [file ijerph-19-09345-s001.zip › Table S1. Search term.pdf]

Low interleukin-12 levels as a main feature of severe malaria: A systematic review and meta-analysis

Polrat Wilairatana[1], Pattamaporn Kwankaew[2], Kwuntida Uthaisar Kotepui[2], Manas Kotepui[2*]

[1]Department of Clinical Tropical Medicine, Faculty of Tropical Medicine, Mahidol University, Bangkok, Thailand

[2]Medical Technology, School of Allied Health Sciences, Walailak University, Tha Sala, Nakhon Si Thammarat, Thailand

*Correspondence: manas.ko@wu.ac.th

PW: polrat.wil@mahidol.ac.th

PK: pattamaporn.kw@wu.ac.th

KU: kwuntida.ut@wu.ac.th

Table S1. Search term

**Databases**	**Search terms/Search strategy**	**Date**
MEDLINE (via PubMed)	(malaria OR plasmodium OR plasmodia) AND ("Interleukin 12" OR IL12 OR IL-12 OR "Interleukin-12")<br><br>Search results: 336	20 February and 2 March 2022
Scopus	(malaria OR plasmodium OR plasmodia) AND ("Interleukin 12" OR IL12 OR IL-12 OR "Interleukin-12")<br><br>Search option: Title, abstract, keywords<br><br>Search results: 736	20 February and 2 March 2022

Embase	(malaria OR plasmodium OR plasmodia) AND ("Interleukin 12" OR IL12 OR IL-12 OR "Interleukin-12")<br><br>Search option: All fields<br><br>Search results: 813	20 February and 2 March 2022
