# Supplementary material for: Low Interleukin-12 Levels concerning Severe Malaria: A Systematic Review and Meta-Analysis
Source: Int J Environ Res Public Health. 2022 Jul 30;19(15):9345. doi: 10.3390/ijerph19159345 (PMC9368085; doi:10.3390/ijerph19159345)
Supplement: Supplementary file 1 [file ijerph-19-09345-s001.zip › Table S3. Quality the included studies.pdf]

Low interleukin-12 levels as a main feature of severe malaria: A systematic review and meta-analysis

Polrat Wilairatana[1], Pattamaporn Kwankaew[2], Kwuntida Uthaisar Kotepui[2], Manas Kotepui[2*]

[1]Department of Clinical Tropical Medicine, Faculty of Tropical Medicine, Mahidol University, Bangkok, Thailand

[2]Medical Technology, School of Allied Health Sciences, Walailak University, Tha Sala, Nakhon Si Thammarat, Thailand

*Correspondence: manas.ko@wu.ac.th

PW: polrat.wil@mahidol.ac.th

PK: pattamaporn.kw@wu.ac.th

KU: kwuntida.ut@wu.ac.th

Table S3. Quality of the included studies

Case-control studies

	**Study**	**Score (out of 22)**	**Score (percentage)**	**Quality**
1.	Chaisavaneeyakorn et al., 2003	18	82	High
2.	Chaiyaroj et al., 2003	18	82	High
3.	Lyke et al., 2004	22	100	High
4.	Malaguarnera et al., 2002	18	82	High
5.	Sinha et al., 2010	19	86	High

Cross-sectional studies

	Study	Score (out of 22)	Score (percentage)	Quality
1.	Nmorsi et al., 2010	17	77	High

Prospective observational studies

	Study	Score (out of 22)	Score (percentage)	Quality
1.	Luty et al., 2000	20	91	High
2.	Perkins et al., 2000	19	86	High
3.	Singotamu et al., 2006	16	73	Moderate
4.	Wroczyńska et al., 2005	19	86	High

STROBE: Strengthening the Reporting of Observational Studies in Epidemiology
